# Symptomatic Aortic Endograft Occlusion in a 70-year-old Male

**DOI:** 10.5811/cpcem.2020.5.46734

**Published:** 2020-07-09

**Authors:** Jose Cardenas, Babak Khazaeni

**Affiliations:** Desert Regional Medical Center, Department of Emergency Medicine, Palm Springs, California

**Keywords:** Emergency medicine, aortic endograft occlusion, leg ischemia, EVAR

## Abstract

**Case Presentation:**

A 70-year-old male with prior aorta endovascular aneurysm repair presented with progressive lower extremity weakness over the course of several hours. There was noted loss of palpable bilateral femoral pulses in the emergency department. Computed tomography angiography revealed a kinked and occluded aortic endograft. He subsequently underwent successful axillobifemoral bypass revascularization.

**Discussion:**

Kinking of endograft limbs and occlusion has been reported in a small percentage of patients. Bilateral leg ischemia due to aortic endograft occlusion is rare.

## CASE PRESENTATION

**Image 1:**
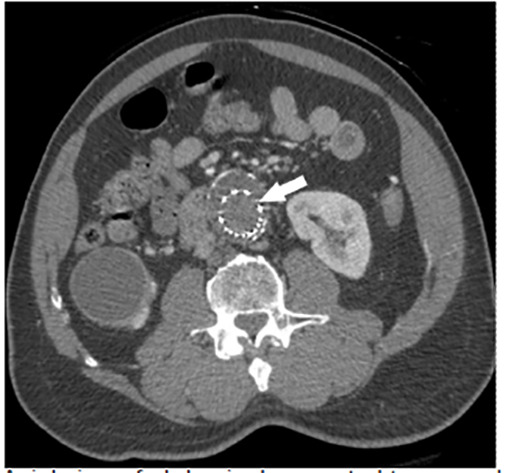
Axial view of abdominal computed tomography angiography demonstrating absence of contrast within the aortic endograft, an indication of occlusion (arrow).

**Image 2:**
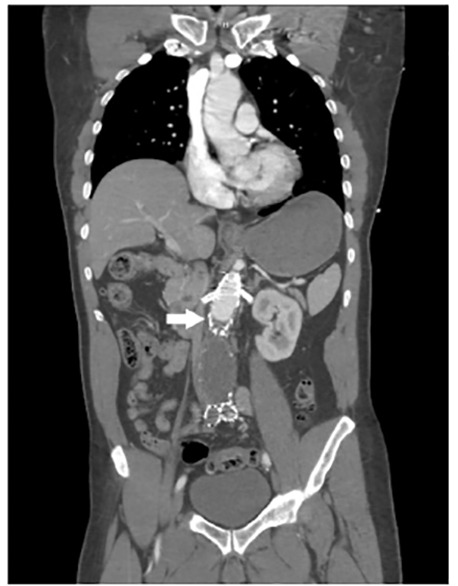
Coronal view of abdominal computed tomography angiography demonstrating contrast reaching but not flowing past the site of occlusion at the aortic endograft (arrow).

**Image 3:**
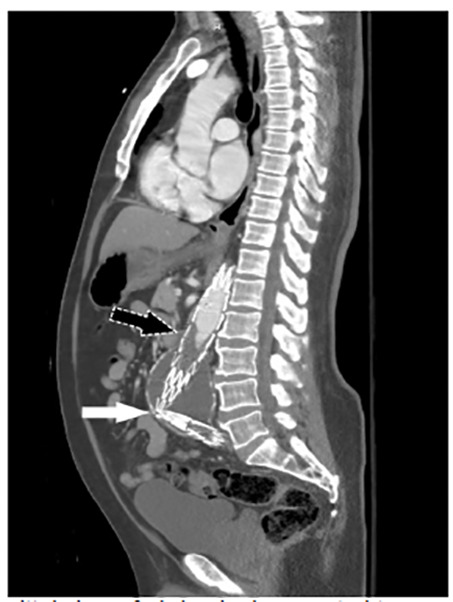
Sagittal view of abdominal computed tomography angiography demonstrating kinking of the aortic endograft (white arrow) and the presence of contrast having reached the location of the thrombus (black arrow) within the endograft.

A 70-year-old male with a history of abdominal aorta endovascular aneurysm repair (EVAR) presented to the emergency department (ED) as a trauma activation after a fall and subsequent lower extremity weakness. The patient reported a near-syncopal episode the night preceding the fall and progressive lower extremity weakness over the course of the morning. On arrival to the ED, he complained of lower extremity weakness with noted initial 2+ palpable, bilateral femoral and dorsalis pedis pulses. Shortly thereafter, he lost palpable femoral pulses bilaterally and had noted cool lower extremities. Computed tomography angiography (CTA) was remarkable for kinking and occlusion of the abdominal aorta endograft below the renal vessels (Images 1–3). The patient was taken to the operating room emergently with successful axillobifemoral bypass revascularization.

## DISCUSSION

The patient had undergone EVAR approximately three years prior and had been taking aspirin and clopidogrel but, per the patient, clopidogrel was recently discontinued by his primary care physician to decrease endoleak at aneurysm repair. CTA demonstrated a kinked endograft and thrombus within the graft into the iliac arteries. Kinking of endograft limbs and occlusion has been reported in 2–4% of patients, which can result in acute limb ischemia.^1^,^2^ Bilateral leg ischemia due to endograft occlusion is rare with a reported incidence ranging from 0%–0.6%.^3^

**CPC-EM Capsule**

What do we already know about this clinical entity?

Kinking and occlusion has been reported as a complication in 2–4% of patients who underwent endovascular aneurysm repair (EVAR) of the abdominal aorta.

What is the major impact of the image(s)?

These images demonstrate kinking and occlusion of the abdominal aorta EVAR endograft with resulting bilateral leg ischemia, which is very rare.

How might this improve emergency medicine practice?

Emergency physicians should consider endograft complications in patients with a history of EVAR presenting with lower extremity neurovascular complaints.
